# Soluble Egg Antigens of *Schistosoma japonicum* Induce Senescence of Activated Hepatic Stellate Cells by Activation of the FoxO3a/SKP2/P27 Pathway

**DOI:** 10.1371/journal.pntd.0005268

**Published:** 2016-12-30

**Authors:** Yinong Duan, Jing Pan, Jinling Chen, Dandan Zhu, Jianxin Wang, Xiaolei Sun, Liuting Chen, Liting Wu

**Affiliations:** 1 Department of Pathogen Biology, School of Medicine, Nantong University, Nantong, Jiangsu, People’s Republic of China; 2 Department of Pathogen Biology and Immunology, Kangda College of Nanjing Medical University, Lianyungang, Jiangsu, People’s Republic of China; 3 Laboratory Medicine Center, Affiliated Hospital of Nantong University, Nantong, Jiangsu, People’s Republic of China; University of Technology, Sydney, AUSTRALIA

## Abstract

**Background:**

Liver fibrosis was viewed as a reversible process. The activation of hepatic stellate cells (HSCs) is a key event in the process of liver fibrosis. The induction of senescence of HSCs would accelerate the clearance of the activated HSCs. Previously, we demonstrated that soluble egg antigens (SEA) of *Schistosoma japonicum* promoted the senescence of HSCs via STAT3/P53/P21 pathway. In this paper, our study was aimed to explore whether there are other signaling pathways in the process of SEA-induced HSCs aging and the underlying effect of SKP2/P27 signal on senescent HSCs.

**Methodology/Principal findings:**

Human hepatic stellate cell line, LX-2 cells, were cultured and stimulated with SEA. Western blot and cellular immunofluorescence analysis were performed to determine the expression of senescence-associated protein, such as P27, SKP2 and FoxO3a. Besides, RNA interfering was applied to knockdown the expression of related protein. The senescence of HSCs was determined by senescence-associated β-gal staining. We found that SEA increased the expression of P27 protein, whereas it inhibited the expression of SKP2 and FoxO3a. Knockdown of P27 as well as overexpression of SKP2 both suppressed the SEA-induced senescence of HSCs. In addition, the nuclear translocation of FoxO3a from the nucleus to the cytoplasm was induced by SEA stimulation.

**Conclusions/Significance:**

The present study demonstrates that SEA promotes HSCs senescence through the FoxO3a/SKP2/P27 pathway.

## Author Summary

Activation of hepatic stellate cells (HSCs) is a key event of liver fibrosis. Induction of activated HSCs apoptosis and inhibition of activated HSCs proliferation are the common anti-fibrotic strategies to block liver fibrosis. The induction of senescence of HSCs is responsible for the clearance of the activated HSCs as well. Senescence of HSCs is mediated by exposure to soluble egg antigens (SEA) of *Schistosoma japonicum* via STAT3/P53/P21 pathway. In this study, we found that SEA induced the senescence of HSCs, accompanied with the increased the expression of P27 protein and the decreased expression of SKP2 and FoxO3a. Either knockdown of P27 or overexpression of SKP2 alleviates the SEA-induced senescence of HSCs. Moreover, SEA droved the nuclear translocation of FoxO3a from the nucleus to the cytoplasm. Hence, the present study demonstrates that SEA promotes HSCs senescence through the FoxO3a/SKP2/P27 pathway.

## Introduction

Liver fibrosis, a major health problem worldwide [1], results from different etiologies of chronic liver injury, and eventually progresses into cirrhosis or hepatocellular carcinoma. Recently, liver fibrosis was viewed as a reversible process [2]. After years of prevention and treatment of schistosomiasis in China, the new cases of *Schistosoma* infection have declined significantly, but there are still thousands of patients suffering from schistosomiasis [3]. The main pathological change of schistosomiasis is the formation of granuloma around the eggs of *Schistosoma japonicum* (*S*. *japonicum*) in the liver, leading to liver fibrosis.

Studies indicate that the activation of hepatic stellate cells (HSCs) is a key event in the process of liver fibrosis. HSCs are activated and then transform to myofibroblasts, once the liver is subjected to stimulations. Activated HSCs synthesize large amounts of extracellular matrix proteins (ECM) such as type I or type III collagen, laminin and fibronectin [4]. In the process of liver fibrosis induced by *S*. *japonicum* infection, HSCs gather around *S*. *japonicum* egg granuloma [5]. Activated HSCs can express a variety of inhibitors of metalloproteinases (TIMPs) to prevent the degradation of matrix proteins, resulting in the replacement of normal liver tissue by collagen matrix and the formation of fibrous scar. Therefore, inhibition of the HSCs activation, proliferation and accelerating the clearance of the activated HSCs are key strategies for the prevention and treatment of liver fibrosis [6].

Substantial evidences support the possibility of the reversibility of liver fibrosis [2]. Recently, studies revealed that with the development of pathologic process, the size of egg granulomas at the chronic phase (12 weeks) and the advanced phase (24 weeks) was smaller than that at the acute phase of *S*. *japonicum* egg-induced liver fibrosis [7]. Researches indicate that the reversion of liver fibrosis is closely related to the increase of the apoptosis of HSCs. Expression of the tissue inhibitor of metalloproteinase-1 (TIMP-1) decreased, and the synthesis of metalloproteinases (MMPs) such as MMP-1 and MMP-13 increased, thereby inhibiting HSCs activation and proliferation, increasing the clearance of activated HSCs as well as the degradation of collagen fiber, and eventually alleviating liver fibrosis [8,9].

Studies showed that the induction of senescence of HSCs would accelerate the clearance of the activated HSCs as well [10]. The senescent cells usually display a cell cycle arrest in the G0 or G1 phase but maintain the metabolic activity [11]. Once senescent, senescence-associated β-gal (SA-β-Gal), the specific maker of senescence, is detected in these cells. In our previous study, we demonstrated that SEA induced the HSCs senescence through the STAT3/P53/P21 pathway [12]. Besides, it has been well established that FoxO3a signaling cascade is implicated in the senescent process of multiple cells [13–15]. It has been revealed that FoxO3a inhibited the senescence of hepatocytes [16]. Additionally, S phase kinase associated protein 2 (SKP2) was reported to suppress cellular senescence induced by oncogenic stimuli independent of ARF/p53 signaling. And cell cycle inhibitor P27, the SKP2 substrate, is targeted by SKP2 for ubiquitination and degradation [17,18]. In this study, we investigate whether the FoxO3a/SKP2/P27 signaling participates in the SEA-induced HSCs senescence.

## Methods

### Reagents

SEA of *S*. *japonicum* were obtained from Jiangsu Institute of Parasitic Diseases (China). SEA was sterile-filtered and endotoxin was removed with Polymyxin B agarose beads (Sigma, USA). Limulus amebocyte lysate assay kit (Lonza, Switzerland) was used to confirm the removal of endotoxins from the SEA as previously described [19]. Primary antibodies for FoxO3a, SKP2, P27 and AKT were purchased from Santa Cruz Biotechnology (USA, antibody dilution for Western blot of all antibodies from this company is 1:200). Primary antibody for phospho-AKT was purchased from Cell Signaling Technology (USA, antibody dilution for Western blot is 1:1000). All of the secondary antibodies were obtained from Santa Cruz Biotechnology (USA, antibody dilution is 1:2000). The staining kit for SA-β-Gal was purchased from GenMed Scientifics Inc (USA).

### Culture of human hepatic stellate cell line LX-2

LX-2 cells, the ‘immortalised’ human HSCs, were provided by Xiangya Central Experiment Laboratory (Hunan, China) and maintained in DMEM with 10% Fetal Bovine Serum in a humidified incubator with 5% CO_2_. Culture medium was replaced every day and cells were subcultured with trypsin when they were at 80% confluence.

### Western blot

Cells were lysed in RIPA cell lysis buffer including protease inhibitor (1mM) and phosphatase inhibitors (1mM). Equal amounts of protein extract were separated by SDS-PAGE and then transferred onto polyvinylidene difluoride (PVDF) membranes. The membranes were blocked in 5% nonfat milk for 2 hours, incubated with the indicated primary antibodies at 4°C overnight, and then incubated with horseradish peroxidase (HRP)-conjugated secondary antibodies for 1 hour at room temperature.

### SA-β-Gal staining

SA-β-Gal staining was performed according to the instruction of SA-β-Gal staining kit, in which cleaning solution, fixation fluid, acidic solution and staining fluid were provided as the main kit contents. Briefly, LX-2 cells were washed with cleaning solution and fixed by fixation fluid for 5 minutes at room temperature. Afterwards, cells were washed by acidic solution twice and stained with staining fluid for 16 hours at 37°C. Finally, SA-β-Gal staining positive cells were assayed using a bright field microscope.

### Immunofluorescence analysis

For the Immunofluorescence staining, cells were seeded in 6-well culture plates and fixed with 4% paraformaldehyde. Afterwards, cells were permeabilized with 0.1% Triton X-100 and then blocked in 5% BSA. After that, cells were incubated with FoxO3a antibody (dilution is 1:50) and visualized with Alexa Fluor 568 conjugated secondary antibody (Invitrogen, USA, antibody dilution is 1:200) under a fluorescent microscopy.

### Vector construction

pcDNA3.1 plasmid was digested with *EcoR*I and *BamH*I (TaKaRa, China), and CDS region of SKP2 (GenBank: NM_005983) was subcloned into pcDNA3.1 vector to generate the recombinant vector pcDNA3.1-SKP2. The recombinant plasmids were verified by restriction analysis and sequencing.

### Cell transfection

LX-2 cells were transfected with P27 siRNA (GenePharma, China) or pcDNA3.1-SKP2 overexpression plasmid by Lipofectamine 2000 reagents (Invitrogen, USA) according to the manufacturer’s instructions. After 24 hours, cells were subjected to various stimulations for indicated time.

### Statistical analysis

Data is expressed as mean ± SEM (standard error of mean) of three independent experiments. All *p* values were calculated using a two tailed paired Student’s t test or a one way ANOVA. *p* < 0.05 was considered as statistically significant.

## Results

### SEA-induced LX-2 cells senescence is related to the P27 signaling pathway

**Fig 1:**
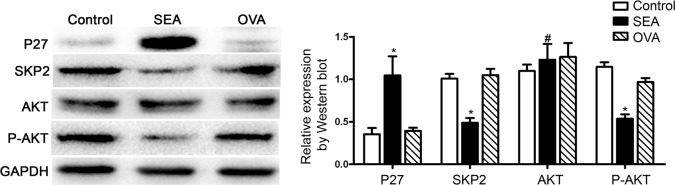
SEA-induced LX-2 cells senescence is related to the P27 signaling pathway. Expression of P27, SKP2, total AKT and P-AKT were assayed by Western blot. * *p*<0.05, compared to control group; # *p*>0.05, compared to control group.

Previously, we found that SEA-induced LX-2 cells senescence via the STAT3/P53/P21 pathway [11]. Since P27, the cell cycle inhibitor, plays an important role in cellular senescence and SKP2 could cause a decrease in the level of P27 expression [13, 20], we next verified whether P27 signaling pathway is implicated in the progress of LX-2 senescence. As illustrated in Fig 1, Western blot analysis showed that SEA markedly increased the expression of P27, but decreased the SKP2 protein level. Furthermore, the expression of P-AKT, the upstream of P27, was also significantly decreased under SEA exposure, although the total expression of AKT was not affected.

### FoxO3a is implicated in the SEA-induced LX-2 cells senescence

**Fig 2:**
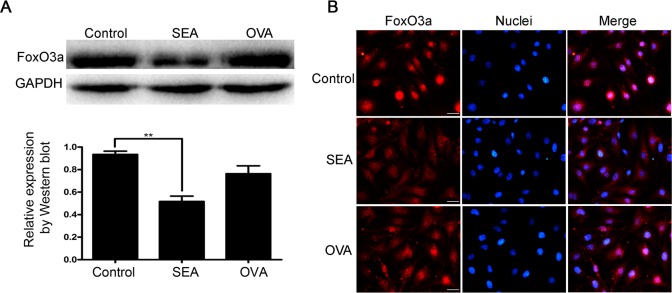
FoxO3a is implicated in the SEA-induced LX-2 cells senescence. (A) The expression of FoxO3a was assayed using Western blot. (B) Nuclear accumulation of FoxO3a was detected by immunofluorescence staining. Compared to the control group, ** *p*<0.01; Bar: 50 μm.

Apart from the regulation of the level of P27 by SKP2, P27 is also regulated by the FoxO3a protein at the transcriptional level [14]. Also, FoxO3a could be regulated by AKT and 14-3-3 protein [21]. Thus, we further investigated whether FoxO3a was involved in the senescence of LX-2 cells induced by SEA. The results of Western blot indicated that FoxO3a was significantly inhibited by SEA stimulation in the LX-2 cells (Fig 2A). Besides, cell immunofluorescence assay confirmed that FoxO3a was transferred from the nucleus to the cytoplasm after SEA treatment (Fig 2B and S1 Fig). These results suggested that FoxO3a was implicated in the SEA-induced senescence in LX-2 cells.

### SEA-mediated LX-2 senescence is dependent on P27

**Fig 3:**
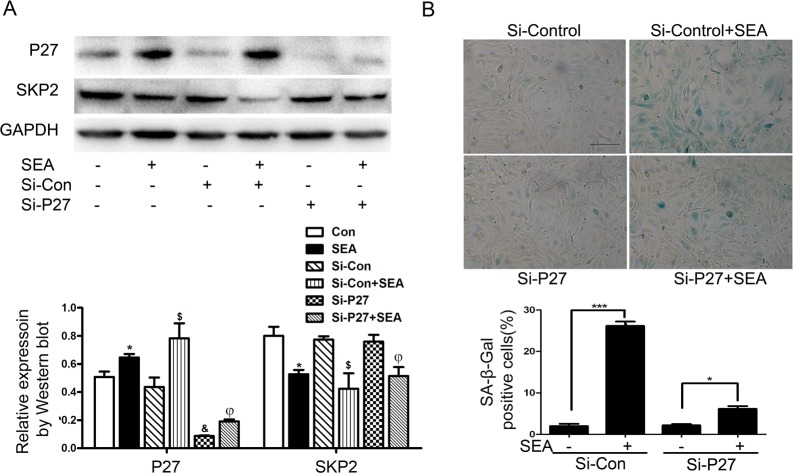
SEA-mediated LX-2 senescence is dependent on P27. (A) The expression of P27 and SKP2 was detected by Western blot in LX-2 cells treated with SEA or P27 siRNA, respectively. * *p*<0.05, compared to control group; $ *p*<0.05, compared to Si-Con group; & *p*<0.05, compared to control group; φ *p*<0.05, compared to Si-P27 group. (B) After treatment of SEA or P27 siRNA, cell senescence phenotype was assayed by SA-β-Gal staining. The proportion of SA-β-Gal staining positive cells was calculated. *** *p*<0.001; * *p*<0.05. Bar: 50 μm.

In order to further verify the role of P27 in the SEA-induced senescence in LX-2 cells, P27 specific small interfering RNA was used to knockdown the expression level of P27 protein in LX-2 cells. As illustrated in Fig 3, the SA-β-Gal staining showed that the senescent LX-2 cells significantly increased accompanied with the upregulated P27 upon SEA stimulation. Nevertheless, the senescence of LX-2 cells induced by SEA was reversed by the P27 siRNA. These results suggest that P27 is a key regulator in the senescence of LX-2 cells induced by SEA.

### SEA promotes LX-2 cells senescence in a SKP2 dependent manner

**Fig 4:**
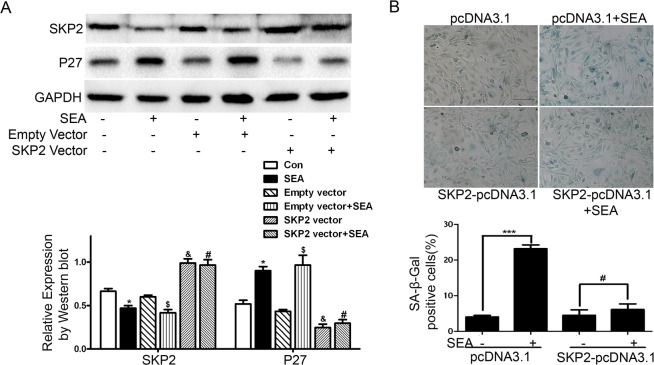
SEA promotes LX-2 cells senescence in a SKP2 dependent manner. (A) The expression of SKP2 and P27 was assayed by Western blot in LX-2 cells treated with SEA or SKP2 over expression plasmid. * *p*<0.05, compared to control group; $ *p*<0.05, compared to empty vector group; & *p*<0.05, compared to control group; # *p*>0.05, compared to SKP2 vector group. (B) SA-β-Gal staining was used to detect cell senescence phenotype after treatment with SEA or SKP2 over expression plasmid, and the proportion of SA-β-Gal staining positive cells was calculated. *** *p*<0. 001; # *p*>0.05. Bar: 50 μm.

Studies indicate that SKP2 plays an important role in the process of cellular senescence [17,20,22], thus, we explored whether SKP2 is a regulator in the SEA-induced senescence in LX-2 cells. We found that SEA inhibited the expression of SKP2 (Fig 1). In order to further investigate the potential mechanism of SKP2 in the process of SEA-induced senescence, specific SKP2 over expression plasmid was constructed and transfected into LX-2 cells, and then the efficiency was confirmed by Western blot analysis. The results showed that the SKP2 protein expression in LX-2 cells was enhanced after transfection with SKP2 over expression plasmid (Fig 4A), and the high expression of SKP2 could inhibit the senescence of LX-2 cells induced by SEA (Fig 4B). These results suggest that SKP2 can inhibit LX-2 cells senescence mediated by SEA.

To explore the mechanism of SKP2 on senescence in LX-2 cells, we also examined the expression of P27, and we found that the expression of P27 in LX-2 cells was significantly restricted after the overexpression of SKP2 (Fig 4A). On the contrary, the expression of SKP2 in LX-2 cells was not affected by the knockdown of P27 expression (Fig 3A).

## Discussion

It has been well accepted that activation of quiescent HSCs is responsible for the excessive production of ECM in liver fibrosis [23,24], and there has been increased recognition in utilizing functions of HSCs for therapeutic applications to reverse liver fibrosis [25]. Thus, preventing the activation of HSCs and increasing the clearance of activated HSCs are viewed as promising anti-fibrotic strategies [4,26,27]. Among these, induction of activated HSCs apoptosis and inhibition of activated HSCs proliferation are the common anti-fibrotic strategies to block liver fibrosis. For example, we found SEA could induce HSC apoptosis and inhibit activation of HSCs under some suitable conditions [19]. In addition, studies showed that the senescence of HSCs would block the development of liver fibrosis. Kong X et al. have demonstrated that IL-22 induced HSCs senescence and restricted the development of liver fibrosis in mice [10,28,29]. Which are different from quiescent HSCs, senescent HSCs often manifest as SA-β-Gal staining positive cells. In the previous study, our results showed that more SA-β-Gal staining positive cells could be found in SEA-treated LX-2 cells and SEA decreased the expression of α-SMA in LX-2 cells partially due to SEA-induced senescence [11].

It has been shown that P53, tumor suppressor protein, plays a critical role in the induction of senescence. We have recently shown that SEA induced HSCs senescence through STAT3/P53/P21 pathway. SEA increased the expression of P-STAT3, P53 and P21. And knockdown of STAT3 or P53 inhibited the SEA-induced senescence of HSCs [12]. Besides the P53-P21 and P16-Rb signaling pathways [30–32], there are other signaling pathways that promoting the development and progression of cellular senescence. The inactivation of retinal vascular tumor suppressor factor (VHL) can decrease the expression of SKP2 and increase the expression of P27, and then induce cellular senescence [33]. Consistent with this result, the overexpression of HTLV-1 Tax protein also reduced the expression of SKP2 and accompanied with the occurrence of cellular senescence in human T cells [34]. These results suggest that the decrease of SKP2 and the induction of P27 might play direct roles in cellular senescence. SKP2 is a member of the F box protein family, and the formation of the SKP2-SCF complex exhibits the E3 ligase activity. Li Z et al. showed that SKP2 regulates cell cycle and cell proliferation by degradation of its downstream molecules such as P27, a cell cycle inhibitor [35–37]. And recent studies have shown that inactivation of SKP2 induces cellular senescence, in which the cell cycle inhibitor P27 and P21 expression are enhanced [17]. Therefore, we suspect that SKP2 is involved in the process of SEA-induced LX-2 cell senescence. We found that SEA markedly inhibited the expression of SKP2, but enhanced expression of P27 (Fig 1). In order to further verify the role of SKP2 and P27 in the senescence of LX-2 cells, we transfected P27 siRNA to LX-2 cells to knockdown the P27 protein expression and transfected SKP2 overexpression plasmid to upregulate the expression of SKP2. These results further confirmed that SEA-induced cellular senescence was partially dependent of SKP2/P27 pathway (Fig 3 and Fig 4).

In addition to the regulation of the post transcriptional level of P27 by SKP2, P27 is also regulated by the FoxO3a protein at the transcriptional level [14]. The data shows that FoxO3a participates in the process of many kinds of cell senescence. Xu-Feng et al. found that FoxO3a can inhibit the senescence of cardiovascular endothelial cells by regulating the cell cycle mediated by ROS [14]. Similarly, in the experiment of Kyoung Kim H et al., FoxO3a also exhibited an inhibition effect on human dermal fibroblast senescence. The experimental results demonstrated that knockdown of FoxO3a could promote the cell senescence [38]. Therefore, we further verify the effect of FoxO3a on the senescence of SEA-induced LX-2 cells, and our experimental results are consistent with the above phenomena. In SEA-treated LX-2 cells, FoxO3a protein expression was significantly inhibited, and FoxO3a occurred nuclear transfer from the nucleus to the cytoplasm under the role of SEA (Fig 2B). Thus, FoxO3a is a key regulator in the SEA-induced senescence of LX-2 cells.

To our knowledge, AKT kinases are critical players in PI3K-mediated signal transduction pathways [39]. AKT phosphorylates downstream substrates to regulate cell growth, proliferation, apoptosis, senescence, and other processes [40]. Cong Fu et al. found that P-AKT expression was down-regulated during the process of cellular senescence induced by H_2_O_2_ [41]. Studies demonstrated that AKT phosphorylated FoxO proteins, leading to the negative FoxO regulation via triggering its nuclear exclusion [21]. In addition, AKT can also promote the degradation of P27 [42]. In the present study, our results showed that the expression of P-AKT was inhibited by the SEA stimulation (Fig 1).

**Fig 5:**
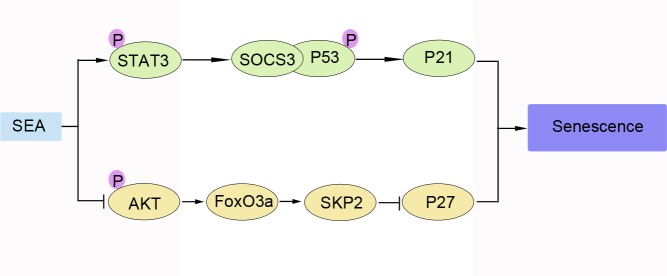
Proposed mechanisms by which SEA could induce senescence in LX-2 cells. SEA could induce senescence in LX-2 cells, partly through STAT3/P53/P21 pathway and partly through FoxO3a/SKP2/P27 pathway.

In conclusion, SEA might slow down the progression of liver fibrosis by promoting HSCs senescence through the FoxO3a/SKP2/P27 pathway. Our previous and present findings provide evidence supporting a possible mechanism by which SEA induces senescence in LX-2 cells (Fig 5) and these provide a potential target of the clinical research of liver fibrosis.

## Supporting Information

S1 Fig FoxO3a is implicated in the SEA-induced LX-2 cells senescence. Nuclear accumulation of FoxO3a was detected by immunofluorescence staining and visualized under oil lens of fluorescent microscopy (original magnification 1000×).
(TIF)
