# Supplementary figures and images for: Soluble Egg Antigens of Schistosoma japonicum Induce Senescence of Activated Hepatic Stellate Cells by Activation of the FoxO3a/SKP2/P27 Pathway
Source: PLoS Negl Trop Dis. 2016 Dec 30;10(12):e0005268. doi: 10.1371/journal.pntd.0005268 (PMC5231384; doi:10.1371/journal.pntd.0005268)

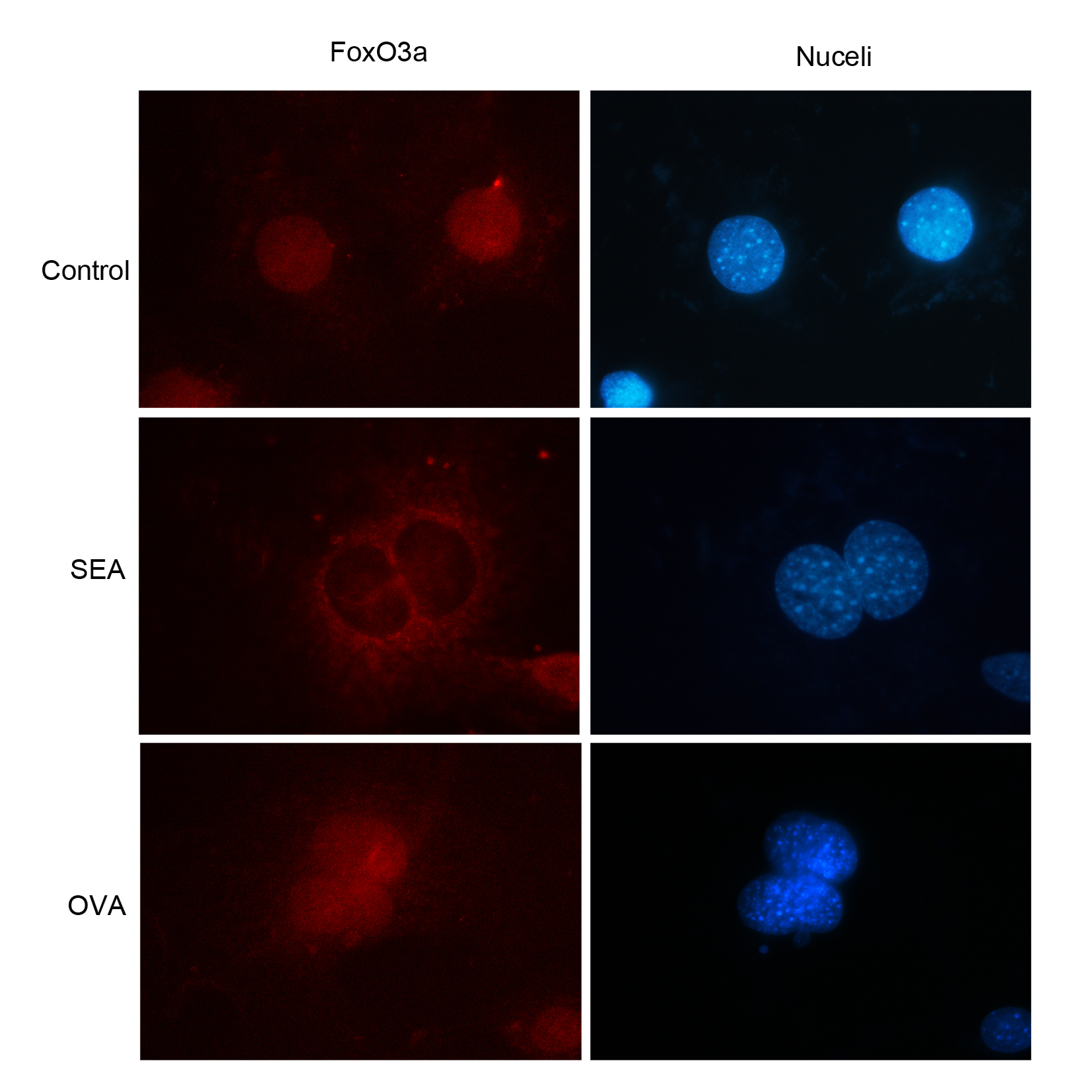

Supplement: S1 Fig — Nuclear accumulation of FoxO3a was detected by immunofluorescence staining and visualized under oil lens of fluorescent microscopy (original magnification 1000×). (TIF) [file pntd.0005268.s001.tif]
